# DeepSurveyCam—A Deep Ocean Optical Mapping System

**DOI:** 10.3390/s16020164

**Published:** 2016-01-28

**Authors:** Tom Kwasnitschka, Kevin Köser, Jan Sticklus, Marcel Rothenbeck, Tim Weiß, Emanuel Wenzlaff, Timm Schoening, Lars Triebe, Anja Steinführer, Colin Devey, Jens Greinert

**Affiliations:** GEOMAR Helmholtz Centre for Ocean Research Kiel, RD4/RD2, Wischhofstr. 1-3, 24148 Kiel, Germany; kkoeser@geomar.de (K.K.); jsticklus@geomar.de (J.S.); mrothenbeck@geomar.de (M.R.); tweiss@geomar.de (T.W.); ewenzlaff@geomar.de (E.W.); tschoening@geomar.de (T.S.); ltriebe@geomar.de (L.T.); asteinfuehrer@geomar.de (A.S.); cdevey@geomar.de (C.D.); jgreinert@geomar.de (J.G.)

**Keywords:** AUV, photogrammetry, camera, survey, deep sea, mapping, mosaicking, Photoscan

## Abstract

Underwater photogrammetry and in particular systematic visual surveys of the deep sea are by far less developed than similar techniques on land or in space. The main challenges are the rough conditions with extremely high pressure, the accessibility of target areas (container and ship deployment of robust sensors, then diving for hours to the ocean floor), and the limitations of localization technologies (no GPS). The absence of natural light complicates energy budget considerations for deep diving flash-equipped drones. Refraction effects influence geometric image formation considerations with respect to field of view and focus, while attenuation and scattering degrade the radiometric image quality and limit the effective visibility. As an improvement on the stated issues, we present an AUV-based optical system intended for autonomous visual mapping of large areas of the seafloor (square kilometers) in up to 6000 m water depth. We compare it to existing systems and discuss tradeoffs such as resolution *vs.* mapped area and show results from a recent deployment with 90,000 mapped square meters of deep ocean floor.

## 1. Introduction

When compared to land or airborne topographical survey methods such as radar, lidar or photogrammetry, hydrographic surveys of the structure and texture of the seafloor are considerably impaired by the body of seawater covering it. Water strongly absorbs a wide range of the electromagnetic spectrum. Penetration distances in clear water are around 100 m for sunlight between 350 nm and 550 nm (blue to green) [1]. Suspended particulates and dissolved organic and inorganic compounds reduce the practically visible range much further [2]. Therefore, seafloor exploration is often executed with single beam (1D), multi beam swath echo sounders (MBES, 2D) and imaging sonars (3D) [3]. In addition to the effects of varying sound velocity due to water body stratification, the spatial resolution of acoustic sounding methods in the deep sea is practically limited to one percent of the slant range. The same rule applies to the positioning accuracy of acoustic underwater navigation devices in the absence of GPS signal reception, posing considerable challenges, particularly for incremental or repetitive surveys in very deep waters [4]. A deep seafloor map may have a local precision of centimeters, but can only be referenced with an accuracy of tens of meters in a global context. Landmarks of known absolute position do not exist and cannot easily be generated by geodetic methods.

These restrictions have led to the development of a suite of long range, imprecise and short range, precise survey methods which play out their combined strengths in a nested survey approach across the entire range of scales required in the deep ocean [5,6]: Regional and reconnaissance surveys are conducted using ship-based acoustic methods (side scan sonar or MBES) which provide an absolute geographic reference frame through the incorporation of GPS positioning and—depth-dependent—resolutions on the scale of tens of meters. Based on this information, autonomous underwater vehicles (AUVs) or deep towed sleds employ the same acoustic methods but closer to the bottom and on a smaller area, increasing the resolution to decimeters. Cabled remotely operated vehicles (ROVs) provide enough maneuverability to carry very close-range acoustics and cameras that resolve down to the millimeter range in highly localized areas. Necessarily, there is a trade-off between resolution and achievable coverage in a given amount of time which is why the nested survey approach requires sequential deployments with each finer resolution survey step being informed by a former one, often spanning several seagoing expeditions.

Beyond the information on seafloor geometry (bathymetry), detailed characterization of lithology, structure and habitat requires information on the texture of substrate and organisms. While acoustic methods can deliver a sort of pseudo-texture (by the strength of the backscattered signal) [7], only optical methods can deliver a full color image equivalent to on-land surveys. In the case of seafloor mapping, color information is predominantly important at small to intermediate scales, while most large-scale features can be assessed using acoustic backscatter that has been verified by local observations (e.g., [8]). Depending on their implementation, optical mapping techniques not only deliver a planar (2D) image mosaic (e.g., [9]) to be draped over an acoustic bathymetrical map but also can directly produce the geometry. This technology is well established in photogrammetry and remote sensing on land, e.g., in aerial mapping of the continents, and comprehensive discussions on technology, survey strategies and mathematical concepts for estimation can be found in standard text books such as the *Manual of Photogrammetry* [10]. In the last two decades, these survey technologies have been extended to largely automated systems relying on machine vision and other sensors, being able to cope with huge amounts of data, GPS-denied environments and other less well defined situations such as unordered photos, camera miscalibration or missing ground control points (*cf.* to [11,12]). A number of approaches have demonstrated the feasibility of AUV-based 3D reconstruction from visual data since then (see for example [13,14,15,16,17,18,19]).

Another way in which current optical and acoustical methods differ is whether they produce geometry extruded vertically from a horizontal plane or a frustum (termed 2.5D, such as by MBES or structured light) or whether they yield a complete three dimensional terrain model, allowing for multiple elevation values for any planar coordinate (*i.e.*, overhangs, cavities) and texture coordinates along vertical walls, cliffs or spires. The latter is a particular advantage of photogrammetric reconstruction from area-scan cameras (either through stereo or structure from motion), which provides the richest data set of all above methods, a three-dimensional model with an intuitively understandable full color texture. Unfortunately, the latter method is also most demanding on water quality and has so far only been applied to close-range surveys in the deep sea (e.g., [20]).

**Figure 1:**
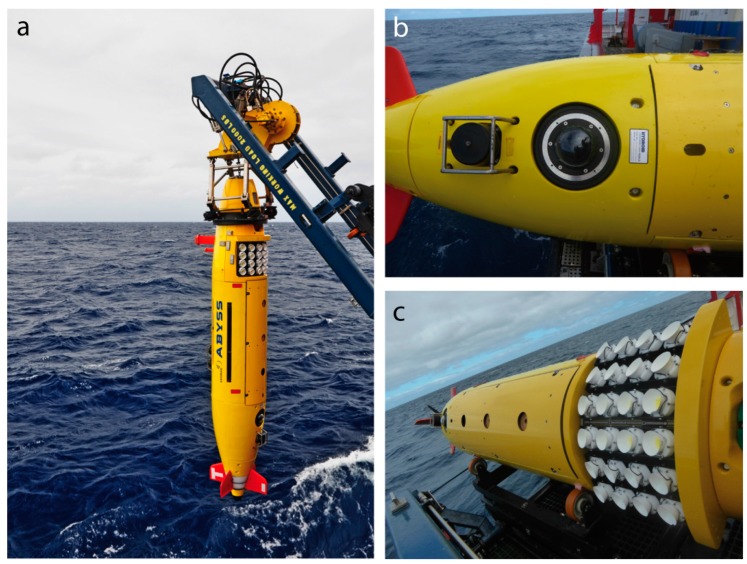
(**a**) The GEOMAR AUV ABYSS prior to launch, equipped with (**b**) a high resolution camera behind a dome port and (**c**) a novel LED flash system.

Despite rapid advances in the development of instrumentation and robotic platforms in recent years [21], a gap has remained in the range of surveying capabilities, that is, an optical system that can cover a large area on the order of 1 km^2^ on a one-day deployment, yielding 3D terrain models at cm-resolution and in full color. We suggest that based on such a model, highly localized observations could be extrapolated with confidence (*cf.* [8]). The larger the coverage of a high-resolution model, the better it can be fit to the absolute geographic reference frame of a low-resolution ship based map, with considerable benefits for time series of studies. These aspirations have led to the development of an AUV based high-altitude color camera system (Figure 1) that allows both for large-scale 2D mosaicking and 3D photogrammetric reconstruction of complex seafloor environments. The details of the system are described below.

## 2. Design Considerations and Previous Work

Situated at a marine research institute, it is important to note that this development has been application driven. The two primary applications behind the design of the system were to map complex morphologies such as volcanic terrain and to efficiently cover large areas e.g., of the abyssal plains in order to conduct large-scale habitat mapping. Necessarily, there is a trade-off between achieved coverage and degree of overlap and (required) redundancy in the data, which may lead to additional dives, depending on the survey strategy. While the surface of flat areas is represented well by a 2D mosaic, for 3D structures with relief more complex representations are required. Although 2D mosaicking is efficient, the distortions induced by high relief (of unknown geometry) bias the scientific evaluations based on such 2D maps, since oblique and sub-vertical areas, cavities or overhangs are poorly or not at all represented. Thus the system would have to be able to supply data suitable for 3D photogrammetric reconstruction. This is based on finding corresponding points in sequences of images in order to compute the (relative) motion of the camera as well as the 3D structure of the environment [11]. While simple geometric statements can sometimes already be made when a seafloor point is observed from two angles, mapping quality is improved and models become denser with a higher number of different observations per point. The simplest hardware setup to achieve this is a single moving camera that takes overlapping images along its way, such that every object is imaged multiple times. Visual reconstruction using a single moving camera, as opposed to using a stereo camera system, requires additional information in order to infer the absolute scale of the scene [11]. In the case of the given system, the AUV’s onboard high precision inertial sensor system that has been reported to drift less than 10 m per hour [22] is utilized.

**Figure 2:**
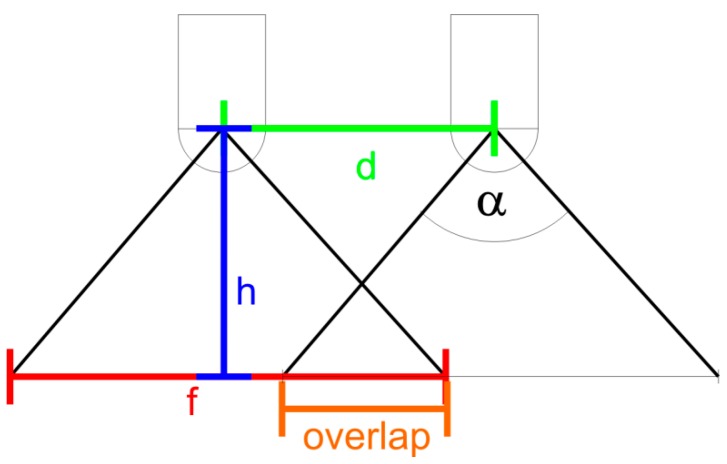
Sketch of image overlap. When a camera with field of view α observes the seafloor from an altitude of h, this creates a footprint of f. After a horizontal movement of d the overlap fraction computes as (f − d)/f, where f can be expressed as 2h × tan (α/2) and d as the product of velocity v and interval t.

The required number of shots depends on the distance, complexity and also the appearance of the observed scene, but in any case no less than 75% overlap should be aimed for in flat terrain. This number has to be further increased e.g., when moving fauna, particles or smoke can partially occlude the view onto the seafloor. Above flat ground, on a moving platform such as an AUV, the overlap fraction (o_along_) can be computed from the image capture interval (t), the vehicle speed (v), the field of view (α) and the altitude above ground (h) according to the following relation:

$$o_{along}=\frac{f-d}{f}=1-\frac{vt}{2h\;\tan\left(\frac{\alpha }{2}\right)}$$

which is illustrated in Figure 2.

Similar considerations can be made for the exposure time. In order to avoid motion blur in the images, the AUV should move less than half the footprint of a single pixel during exposure. This footprint (*f*_pixel_) can be computed from the size of a pixel on the sensor (*p*), the focal length of the lens (*l*) and the altitude (*h*) by the intercept theorem as:

$$f_{pixel}=\frac{p}{l}h$$

Common DSLR cameras currently have typical pixel sizes of *p* = 6 µm although novel sensors with larger pixels are available. This is also a limiting factor for the obtainable accuracy of 2D and 3D surveys. A wide-angle lens of α = 100° field of view has a focal length of *l* = 15 mm when using a full-frame sensor. This would lead to a pixel footprint of 4 mm at *h* = 10 m altitude and respective footprints when varying the parameters. The footprints of rectilinear lenses do not change for pixels in the image center or towards the boundaries, those of equiangular fisheye lenses become larger towards the image boundary. Consequently, in downward looking fisheye images above flat terrain motion blur will first be visible in the image center whereas the motion blur is independent of the image position when using a rectilinear lens.

This illustrates that it is attractive to maximize the field of view in order to both cover a large footprint with every image and secure across-track overlap but also to cast oblique rays onto strongly undulating relief which would obstruct the view of a narrowly downward looking camera.

Assuming a carrier AUV that moves at a certain speed (*v* = 1.5 m/s), and targeting at an overlap of more than 75% along track, we arrive at the following constraint:

$$h\;\tan\left(\frac{\alpha }{2}\right)>3.75\frac{m}{s}t$$

When operating the system using a flash, the recharge time for the flash provides a lower bound on the photo interval t. For instance, when flashing with 1 Hz, the minimum altitude is 3.75 m at a field of view of 90°, or 6.5 m at a field of view of 60° or 14 m for 30°.

For simplicity of presentation in the following we assume the same field of view and footprint width along track and across track. When the AUV goes on a straight line, the covered area A_line_ can be approximated (for readability of equations we will ignore the time taken for the extra “no-overlap” area of the first and the last image, as well as of the first/last line in a pattern and rather consider the asymptotic case of very long and many lines, where these constants can be safely ignored) from the product of the (across-track) footprint and the distance traveled:

$$A_{line}=2h\;\tan\left(\frac{\alpha }{2}\right)vt$$

When the survey area is scanned using a pattern of overlapping parallel linear tracks (commonly called a “lawnmower pattern”), the area is reduced by the across-track overlap o_across_ between two subsequent lines. The goal of having this overlap is to generate seamless maps without holes even in presence of small navigation drift, but also to find back corresponding points from the previous line in order to perform re-navigation (bundle adjustment). We suggest having at least 33% overlap.

The maximum coverage achievable in a given amount of time is given by the following relation:

$$A_{area}=2h\;\tan\left(\frac{\alpha }{2}\right)vt(1-o_{across})$$

From this it can be seen that a large field of view is the enabling property for large-scale optical surveying. Cameras for the deep sea have to be mounted in pressure housings that can resist the approximately 600 bar at 6000 m water depth. The camera views the outside world through a port and these ports are typically flat or spherical. Flat ports for these depths have to be several centimeters thick, depending on the diameter of the opening. Refraction at the port will however change the imaging geometry substantially and in particular invalidate the pinhole camera model [19]. Additionally, refraction limits the maximum field of view theoretically possible, e.g., of a fisheye lens behind a flat port, to roughly 96°, and in practice a field of view above 70° is very difficult to realize using a flat port. Dome ports do not limit the field of view and do not cause refraction for the principal rays if the entrance pupil is aligned with the dome port center. However, they act as a lens themselves, shifting focus very close such that lenses behind dome ports have to use an adapted focusing strategy.

A further challenge in underwater imaging is scattering and absorption [1], both of which are complex physical phenomena. The loss of “signal flux” can however be approximately described by an exponential relation:

$$E\left(x,\lambda \right)=E\left(0,\lambda \right)e^{-\eta \left(\lambda \right)x}$$

where E(0) is the irradiance of (parallel) light at some position and E(x) is the attenuated irradiance after the light has travelled a distance of x through the water. η is the wavelength-dependent attenuation coefficient that represents the loss of flux due to absorption and scattering. When viewing an object with a camera, this remaining exponentially attenuated signal is now additionally degraded by light scattered into the viewing path that reduces the signal to noise ratio. Since this scattering happens largely in direction of 180° [1] it is advisable to move the light source away from the camera, such that direct light scattering into the image is avoided. Still, image quality degrades exponentially with altitude, in particular when both the light source and the camera are moved further away from the target.

This implies that: (a) the light source and the camera have to be developed in interdependence of each other and should be separated as far as possible (to limit scattering in the immediate front of the camera) and (b) a wide field of view is problematic, since the light has to traverse a considerably larger distance in the case of peripheral rays, subjected to scattering and absorption. Therefore, an optimum has to be found between the lighting (geometry and wavelength), field of view and survey geometry dictated by the capabilities of the vehicle.

Development of existing deep ocean camera systems and illumination devices has been primarily oriented on the platform they are deployed on. In order to illustrate the spectrum of technical realizations of the optical mapping task, Table 1 gives a non-exhaustive overview of systems developed throughout the past two decades.

The AUV ABYSS at GEOMAR is a type REMUS 6000 flight-class AUV manufactured by Hydroid, Inc. (Pocasset, MA, USA) (Figure 1). It is 4 m long and has a torpedo like shape at a maximum diameter of 0.66 m.

**Table 1:** A non-exhaustive selection of ocean floor imaging systems of the last two decades reveals the spectrum of applications and solutions, including the original and new camera configurations of the GEOMAR AUV ABYSS. Abbreviations for methods are (M) mosaicking, (SL) structured light, (P) photogrammetry, either using stereo or structure-from-motion. Measures of efficiency are hard to normalize due to non-uniform information on actual image overlap e.g., during nonlinear vehicle tracks. Therefore, the (FoV) field of view, (V) velocity over ground, (t) survey duration or (A) total area covered are reported.

Year Deployed	Research Group, Vehicle	Vehicle Category	Method	Camera Model	Imager	Mode	Depth (m)	Altitude (m)	Cadence (s)	Ground Resolution (mm)	Efficiency	Reference
1996	WHOI, Argo II	Towsled	M	Marquest ESC9100	mono	b/w	1600	10	13	15	3152 m^2^/h	Escartin *et al.*, 2008 [23]
2007	GEOMAR, Abyss	AUV flight	M	AVT Pike	mono	b/w	6000	4–12	4	6	FoV = 42 × 42°, V = 1.5 m/s	this study
2008	NTNU	ROV	M	Uniqvision	mono	color	500	1.9	4	2.2	800 m^2^/h	Ludvigsen *et al.*, 2007 [24]
2008	Ifremer, Victor	ROV	M	OTUS	mono	b/w	6000	8–10	10–17	10	6491 m^2^/h	Prados Gutierrez *et al.*, 2012 [25]
2010	URI, Hercules	ROV	SL/P	AVT Prosilica	stereo	color	2000	3	6.7	2.5	FoV = 35 × 52°/V = 0.15 m/s	Roman *et al.*, 2010 [26]
2010	WHOI, Seabed	AUV hover	P	Pixelfly	mono	color	2000	3	3	\	A = 30 × 45 m/V = 0.25 m/s	Bingham *et al.*, 2010 [27]
2011	Kongsberg, HUGIN	AUV flight	M	Tilecam	mono	b/w	1000	2–5	<1	2.3	FoV = 55 × 35°, V = 2 m/s	Hagen, 2014 [28]
2013	Univ. Tokyo, Hyper Dolphin	ROV	SL	SeaXerocks 2	mono	color	2000	13	5	6.4	8144 m^2^/h	Bodenmann *et al.*, 2013 [29]
2013	Univ. Girona, Girona 500	AUV hover	P	Canon 5d Mk II	stereo	color	500	3.68	2	0.66	t = 103 min, A = 65 × 20m	Gracias *et al.*, 2013 [30]
2014	NOC, Autosub	AUV flight	M	Point Grey Grasshopper	mono	color	6000	3.2	0.87	0.59	23,000 m^2^/h	Morris *et al.*, 2014 [31]
2015	ACFR, Sirius	AUV hover	P	AVT Prosilica GC1380	stereo	color	800	3	0.67	2.2	FoV = 45 × 38°, V = 0.5m/s	Williams *et al.*, 2015 [32]
2015	GEOMAR, Abyss	AUV flight	P	Canon 6D	mono	color	6000	4.7–12	1	1.9–5	25,700–166,000 m^2^/h	this study

**Figure 3:**
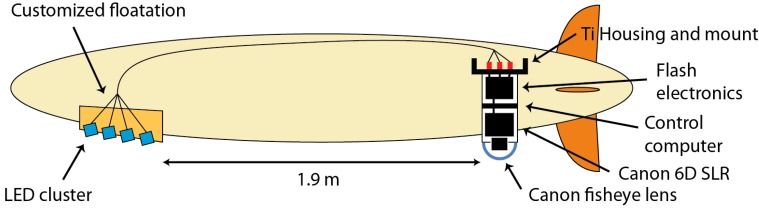
Schematic overview of the components of the newly designed high-altitude camera system for the GEOMAR Remus 6000 AUV.

The AUV has a practical endurance of 16 h, or 100 km and provides a maximum constant hotel power of 28 V, 6 A available to scientific payload systems. It is dynamically steered by two perpendicular pairs of stern fins, which require a nominal speed of 2.5–3.5 kn. Navigation close to the seafloor is carried out by dead reckoning using a Kearfott inertial navigation system aided by a Doppler Velocity Log, and is subject to a drift of less than 10 m [22] per hour. Additionally, long baseline (LBL) transponder fixes can be incorporated if necessary. An altimeter allows following the terrain contours at a fixed altitude, and a forward-looking pencil beam detects obstacles in the way. Due to the high speed and limited obstacle avoidance capabilities, its main strength lies in acoustic mapping several tens of meters above ground, but navigation closer than 8 m to the seafloor is only possible in well charted, rather level terrain. Payloads can be integrated as part of customized floatation foam segments in the lower bow section (sensor bay) and in a downward oriented cylindrical space located towards the stern of the vehicle (processing electronics). This provides a hard design constraint towards a single light source and a single camera. The original imaging system that was part of the standard sensor package uses a 200 joules xenon flash capable of a 0.5 Hz repetition rate. An Allied Vision Pike b/w, 4 MP camera delivers images with a 41° field of view (FoV). Furthermore, we found the dynamic range of the camera insufficient for the production of complex photographic maps. Given the circumstances we decided for a complete redesign of the camera and lighting unit (Figure 3).

Evaluating the strengths and weaknesses of other systems and with the given constraints of our carrier platform, the design goals were the following:

1. An operating altitude of 5–15 m due to limited maneuverability, requiring strong lighting and a sensitive imager;
2. Provisions to maximize the covered area (e.g., high capacity data storage), in order to create large-area seafloor maps in a single deployment;
3. An image acquisition rate of 1 Hz or better to guarantee multiple (>4) perspectives of complex objects;
4. Color still frame sequences at 12 bit or better, resolving ground features smaller than 1 cm, including automatic adaptation to changing seafloor brightness;
5. A wide FoV on individual images to maximize along- and across track overlap, to compensate for small errors in vehicle navigation running parallel track lines and to cast oblique peripheral rays imaging sub-vertical seafloor features in spite of a vertically downward mounted camera orientation;
6. Adhering to the vehicle’s overall depth rating, a pressure resistance of 600 bar plus safety margin.

## 3. Description of Hardware Components

The overall design can be seen in Figure 3 and is outlined in the following paragraphs*.*

### 3.1. Lighting

The strong required illumination ruled out the use of constant lighting in favor of pulsed lighting, so it was decided to use a cluster of 24 LED arrays (Figure 1c). A color temperature of 5600 K was chosen to minimize absorption. By optimizing the power management in pulsed operation mode we were able to increase the light efficiency to 1.6 times the nominal value stated by the manufacturer. Each two arrays are switched in serial configuration, forming 12 parallel pairs driven by capacitor based flash electronics in the rear half of the camera housing.

A 4 Hz maximum operating frequency was found to be a good compromise of image cadence *versus* available power. In order to accommodate the comparatively fast velocity of the AUV, flash durations were kept short and can be varied between 1 ms and 4 ms. A variable trigger delay of 200 μs–12 ms can be manually preset accommodating a variety of cameras. The cluster of 24 individual light sources was mounted flexibly in six rows of four lights, to allow for customized beam forming of the illumination cone. Each light can be tilted individually along the longitudinal vehicle axis and every set of four lights that each can be tilted laterally. This setup allows reacting flexibly to different camera optics or peculiarities of the terrain and visibility conditions. Each LED array is equipped with an external reflector casting a circular light cone with a 74° full width half maximum opening. Using a patented procedure [33], each LED waver is encased in a transparent, pressure neutral resin cast that effectively retains the in-air optical properties of the waver-reflector pair. This solution is not only extremely cost effective, but also light (45 g in water per unit), robust, mass producible, and has since found application in various other GEOMAR instruments. Provided the lights are operated in water, no thermal issues were found.

### 3.2. Optical System and Housing

**Figure 4:**
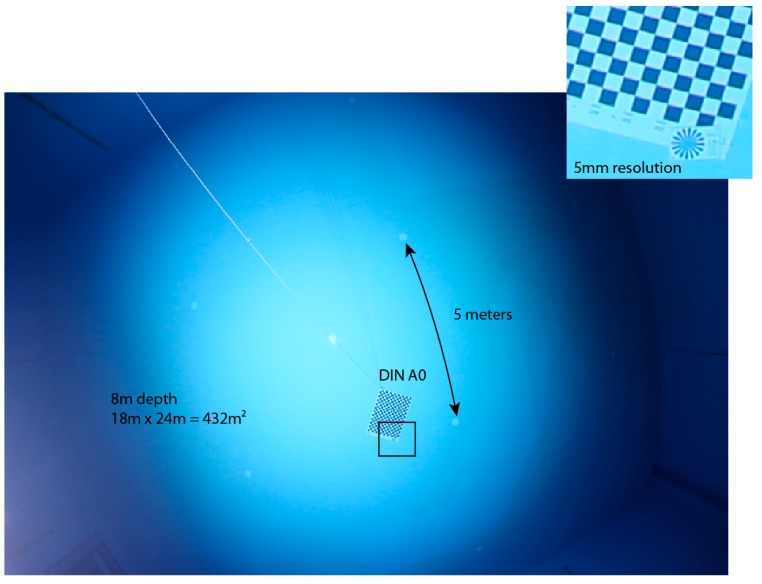
An unprocessed sample image of the system in the test pool of the German Center for Artificial Intelligence (DFKI) in Bremen shows the illumination pattern and achievable resolution in clear water conditions. The inset image shows an enlarged portion of the resolution target in the image center.

In order to maximize the field of view not just for AUV use but possibly also for ROV deployments of the new camera system, the Canon 8–15 mm f4 fisheye zoom lens was implemented (Figure 4), offering a very wide range of FoV settings without having to change the lens. The fisheye lens design by itself produces a curved focal surface that complies with the dome port optics, and generally offers a large depth of field. In order to keep image brightness and depth of field at a compromise, aperture is set at an intermediate value of f8. In order to match the fisheye FoV, a new camera housing was designed which features a dome port with 100 mm inner diameter and 7.1 mm wall thickness, offering a FoV of 160°. The housing dimensions were maximized to 182 mm inner diameter in order to accommodate the flash and camera electronics.

### 3.3. Camera System

Due to the large object distance and limited power supply, the foremost design criterion was the sensitivity of the imager. Following a series of sensitivity tests with machine vision cameras (which are commonly used in deep sea applications), the Canon EOS 6D full frame SLR camera was selected since it is small enough to fit into the pressure housing with minimal modifications. This choice yielded the best immediately available image quality and avoids image post processing procedures as often required with raw machine vision data. A major drawback of SLR cameras is that they have moving parts (the shutter and mirror mechanics), which are prone to wearing off. In particular, according to the manufacturer the shutters have an endurance of 100,000 exposures, and indeed the shutter of our first test system broke after little more than this figure. Consequently, three spare cameras are kept and proactively exchanged for each cruise, with an average dive producing up to around 40,000 images. All cameras are serviced after a cruise.

The camera is linked to a miniaturized PC workstation through a USB2 connection. Contrary to the original camera system, this new controller is completely autonomous from the AUV and does not receive or send any data in order to avoid interference with the vehicle operations. Accessible over gigabit Ethernet through an external port on the housing, all camera settings can be pre-programmed and scripted through custom developed software employing the Canon Software Developers Kit (SDK). A freely configurable intervalometer can be triggered either by a timer or by depth readings received through a pressure sensor integrated in the camera housing, avoiding unnecessary exposures during descent and ascent. Several different missions can be scheduled in a queue. The aperture is constrained by depth of field considerations on the one hand and the limited available light on the other hand. As there is absolutely no light when the flash is off, the effective exposure time is bound to the flash duration. Consequently, only the ISO sensitivity (amplification) of the sensor can be adjusted to react to brighter or darker environments or to varying distances. Since the inbuilt light metering of the camera does not work (as between the photos the flash is recharging), another way of controlling image brightness is required. As we aim at significantly overlapping photographs (75% and more) it is reasonable to assume that only the topmost small stripe of the image will depict a novel portion of the seafloor, which even often consists of terrain with similar color as in the current image.

Consequently, the brightness histogram of the current image can be exploited for adjusting the ISO setting for the next image, in a way that the dynamic range of the next image will be covered well, but that the image should not be overexposed: In the (most relevant) center region of the image we sort all pixel values and require that the 90th percentile be below 90% of the saturated value (*i.e.*, for 8 bit with maximum pixel value 255, we test whether 90% of the pixels are below 230) and adjust the ISO speed for the next image accordingly. See Algorithm 1 for details.

**Algorithm 1.** ISO speed determination for next image based on histogram of current image. For presentation reasons the algorithm is oversimplified. ISO steps are not only available as factors of 2, as assumed in the algorithm above, but sometimes also at intermediate steps. We check whether the next ISO speed is likely to still be within the acceptable range, and so the factor of 0.5 resp. 2 in the above has to be exchanged with “factor to next ISO speed”.

1. Compute histogram of pixel values
2. Determine 90% quantile: q_90_
3. if (q_90_>0.9*MAX_PIXEL_VALUE)
 // image is oversaturated / too bright, lower ISO !
 ISO-speed_t+1_ := 0.5 * ISO-speed_t_
 else if (2*q_90_<0.9*MAX_PIXEL_VALUE)
 // image is dark, even with the next higher ISO image would not be oversaturated
 ISO-speed_t+1_ := 2 * ISO-speed_t_
 else
 // image seems ok, do nothing
 ISO-speed_t+1_ := ISO-speed_t_


In practice, the maximum cadence is limited by the USB2 connection between camera and PC and by restrictions imposed by the Canon SDK, resulting in 1 Hz and 0.5 Hz frequencies for full resolution jpeg and raw images, respectively. The images are written to a 1 TB solid-state hard drive, which is exchanged or copied over network after the deployment.

### 3.4. Calibration

**Figure 5:**
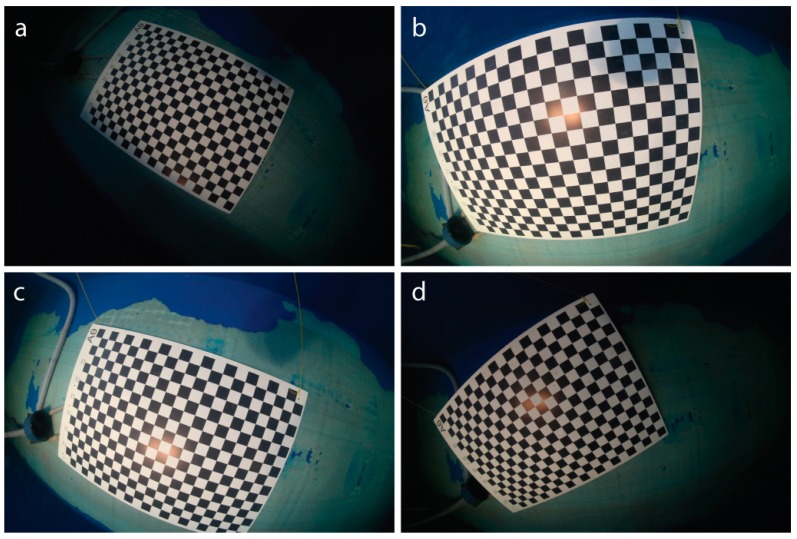
Sample images used in calibration of the camera. The checkerboard is presented in different positions and orientations (**a**–**d**) relative to the camera in order to obtain the 3D ray associated to each pixel in the image.

So far the camera system is integrated into the AUV such that the camera moves up when the AUV moves forward, and is aligned to the vehicle coordinate system up to a mechanical precision of approximately 1°. Although cameras with fisheye lenses often exhibit a caustic, we approximate the lens as having a single center of projection. It is then mounted such that its nodal point coincides with the dome port center, such that the principal rays are not refracted and the pinhole camera model can be used. For calibration, a one square meter calibration pattern (DIN A0) [34] is presented in 20 different orientations and we obtain the coefficients of the fisheye lens using the OCamCalib [35] software (Figure 5).

## 4. Field Trials and First Results

**Figure 6:**
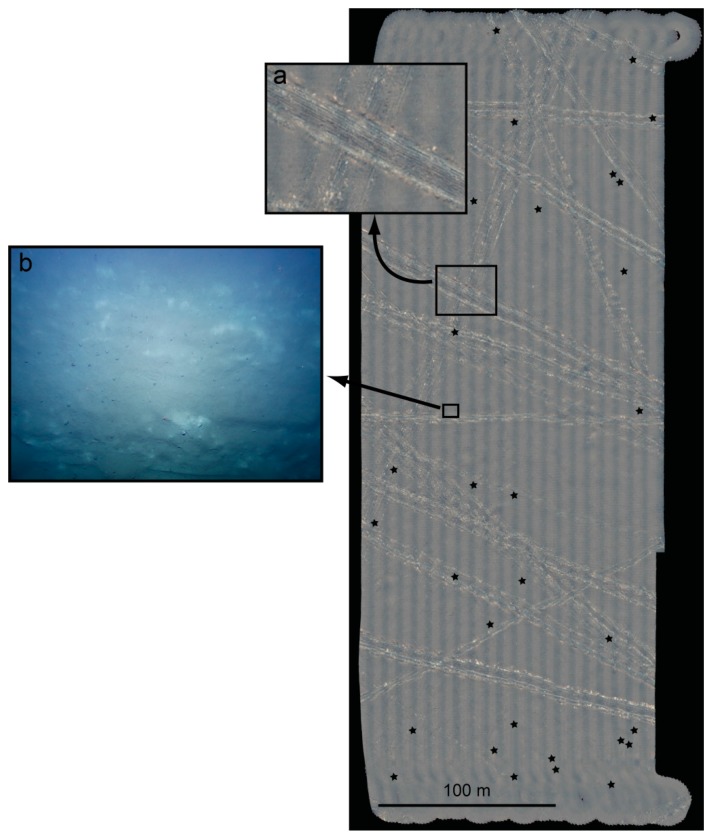
An area of approximately 200 m × 450 m in the DISCOL experimental area of the south-east Pacific ocean offshore Peru. The photo-mosaic consists of 13,000 photos taken from an altitude of 4.7 m on average, captured during 3.5 h by the novel camera system of GEOMAR’s AUV Abyss in 4100 m water depth. The tracks are 8 m wide plowmarks (**a**) from a 1989 experiment to simulate deep sea mining and they are well visible in 2015. The resolution of the images captured (**b**), undistorted but not color corrected) allows to systematically evaluate megafauna that recolonized the area. Asterisks mark the positions of manual offset measurements.

The camera system was deployed on two cruises of the German research vessel SONNE to the Pacific Ocean in 2015 (21 photo dives during SO-239 and SO-242 with approximately half a million photos in total). These scientific cruises took place in the context of the JPI Oceans project initiative “Ecological aspects of deep sea mining”, which investigates potential impacts of (manganese nodule) mining from the abyssal planes of the Pacific Ocean. The sample imagery of Figure 6 has been captured during cruise SO-242-1 to the DISCOL experimental area offshore Peru. Here, a long-term disturbance experiment has been started in 1989, where an 8 m wide plow had been towed through the seafloor for several weeks, in order to simulate mining activity. Now, after 26 years, the same location has been revisited and the entire area has been mapped using acoustic and visual sensors, in particular also using the AUV camera system discussed in this article. Among other things the desired analyses target the change of the habitat and its potential recovery, the time taken for recolonization, and the risk for extinguishing certain species. Such analyses should be supported by a broad data basis as they might later be used for developing guidelines for future mining activities in the deep sea.

A number of post processing approaches have been considered and are still subject to refinement. Figure 6 shows an area of approximately 200 m × 450 m that was mapped in 3.5 h on one of the AUV dives in 4135 m water depth flying at a target altitude of 4.7 m. As a proof of concept, the mosaic has been created by stitching together the individual photographs using navigation data only: Each image was undistorted and empirically normalized using a robust average image that captures vignetting, illumination and attenuation effects. The resulting image was projected onto a plane at 4135 m water depth using the intrinsic camera parameters obtained by calibration and the AUV’s navigation (altitude, pitch, longitude and latitude) as exterior orientation. While the absolute positioning accuracy is on the order of 50 m (tied to the water depth), the local fit between tracks (depending on the dead reckoning algorithm of the AUV) is typically better than 2.5 m as determined by 30 manual random spot measurements (see Figure 6). This is not accurate enough for quantitative studies yet sufficient for timely decisions on further actions at sea. The technique allows for rapid results on the ship and generates the mosaic in a few seconds per input image using a multi-band blending strategy (similar to [36]). The result of rapidly merging more than 10,000 images is a virtual seafloor mosaic as one would observe without water from several hundred meters altitude: The 26 year old tracks are still clearly visible and the visual map provides an excellent overview of the situation in the target area. When zooming into the mosaic or looking at individual images on the other hand, the mega fauna that has recolonized the area is clearly resolved.

**Figure 7:**
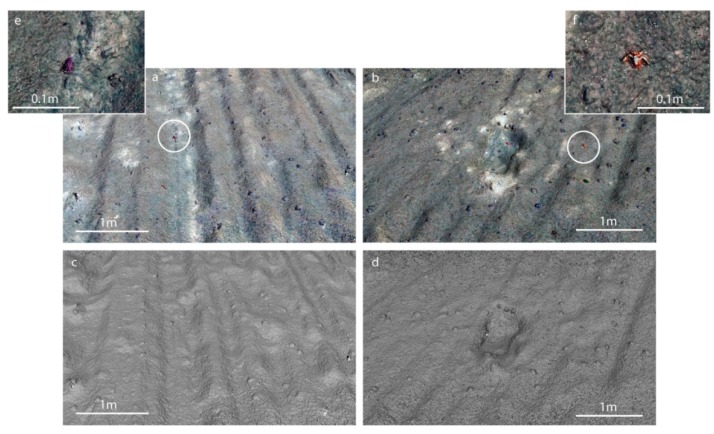
Photogrammetric dense point cloud reconstruction delivers geometry with approx. 1 mm resolution from an altitude of 4.7 m. Renderings show (**a**) artificially perturbated sediment with manganese nodules and holothurian and (**b**) perturbated sediment and the excavation footprint of a box corer. (**c**,**d**) Show the shaded relief of (**a**,**b**), respectively, while (**e**,**f**) show details of epibenthic organisms (white circles) depicted with their rough geometrical shape. Point density equals about one per 2 mm. Compare Figure 6b for a source image of areas (**b**,**d**).

It is also possible to carry out 3D reconstruction from the images (*cf.* to [37] for details on 3D reconstruction from underwater images). As a proof of concept we have processed two test subsets of roughly 10 images from the DISCOL dataset in the commercial software AgiSoft PhotoScan Pro and obtained the 3D models as displayed in Figure 7.

**Figure 8:**
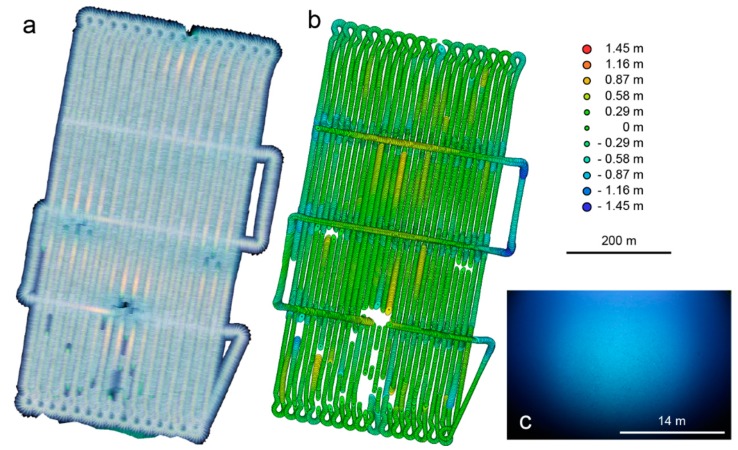
Photogrammetric Reconstruction of a manganese nodule field in the Clarion Clipperton Fracture Zone (CCZ) surveyed during SO239. (**a**) shows the color corrected mosaic without brightness correction to reveal tracks. Colored ellipses in (**b**) mark the extent of lateral positional deviation relative to the acoustic vehicle navigation; the color legend marks the vertical deviation; (**c**) shows a raw fisheye image midway through the survey. Scale bar applies to image center.

Scaling and referencing was again carried out using the vehicle navigation record, which provides the motion baseline between adjacent images in the structure from motion reconstruction step (e.g., [38]). The resolution from 4.7 m altitude is sufficient to reconstruct individual manganese nodules, the profile of the trawl-marks and even the geometry of some of the animals. As this article focuses on the design of the camera hardware and since the optimization of the post processing workflow is still under development, a detailed comparison and evaluation of different photogrammetric reconstruction software packages is out of scope of this article. The results demonstrate however that the overlap and the image quality are sufficient for recovering geometry at sub-centimeter resolution. In order to evaluate the feasibility of this reconstruction workflow on the scale of an entire survey and to judge the quality of referencing, an earlier data set of a similar manganese nodule field from the Clarion Clipperton Fracture Zone surveyed during the SO239 cruise was processed (Figure 8).

It was chosen because the AUV was flown at an altitude of 9 m, which was at the brink of visibility. At 10 m line spacing and an image cadence of 1 Hz an area of 350 m × 700 m was covered. Using Photoscan Pro, an area of 0.24 km^2^ was reconstructed from 9635 images with an average nine-fold overlap. The root mean square positional error (*i.e.*, the offset between the original vehicle navigation and the internally consistent camera pose estimation) as illustrated in Figure 8b is 2.96 m (see Table 2 for further statistics, at an average reprojection error of less than 0.6 pixels). This is an error of less than 1 percent with respect to the extent of the survey area and suggests that the scale is correct. At this point accuracy cannot be further improved without known scale and position of seafloor features, which are generally unavailable in our study areas.

**Table 2:** Statistical offset in meters between acoustically determined navigation and photogrammetrically reconstructed camera poses.

Statistical Measure	Total	X	Y	Z
**RMS Error**	2.96	1.30	2.65	0.28
**Median**	2.83	0.09	−0.15	0.01
**Minimum**	0.02	−6.29	−5.33	−1.45
**Maximum**	7.77	4.78	7.44	0.76

## 5. Discussion

**Figure 9:**
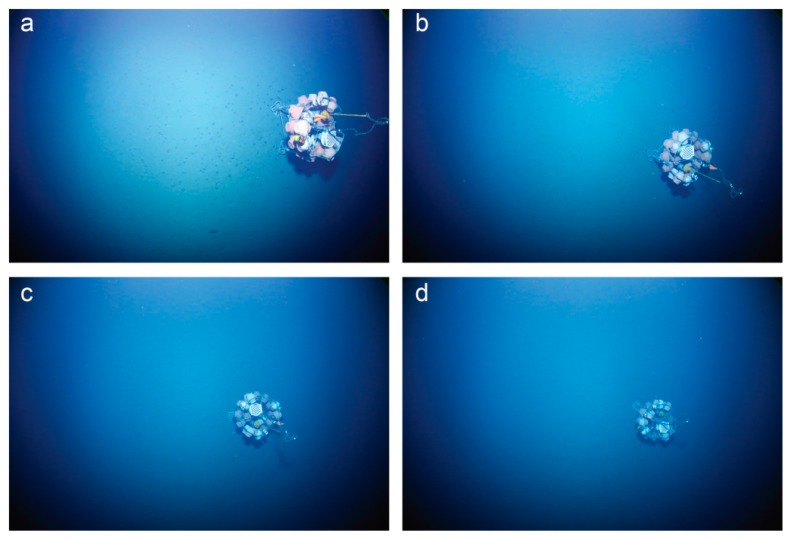
Cropped and undistorted photographs of an autonomous benthic lander (3 m high, 4 m in diameter) photographed from an altitude of (**a**) 6 m; (**b**) and (**c**) 8 m and (**d**) 10 m. Loss in color and light scatter are clearly increasing together with altitude.

The field results show that the system is capable of capturing image data at altitudes from 4 m to 9 m. Compared to the original design goals stated in Section 2, most (such as high resolution, wide field of view and depth rating) were met although our design fell short of some aspects during field tests, foremost the highest possible survey altitude. Figure 9 presents images of the same object at different altitudes. The lowest altitude produces the smallest footprint and the highest resolution image material. Altitudes below 7 m contain color information; above 7 m the red channel is lost.

At least for the Peru basin and CCZ, the initially desired altitude of 15 m thus proved to be too high to yield useable results, although other parts of the ocean may yield better visibility. Small gaps in the southern half of the SO239 survey shown in Figure 8 are due to 3 m deep depressions that increased the camera distance to the ground beyond a maximum acceptable threshold of 9 m, resulting in alignment failure of the respective images. This defines the local maximum working distance determined by water clarity.

We draw the conclusion that realistic working distances of 4–7 m yield color texture information while scans of mere seafloor geometry can still be carried out up to a height of 10 m under optimum conditions. A further challenge is the high amount of light scattered back from the water, an effect that was substantially stronger on the test deployments than in the (actively filtered) test tank. This may require a re-evaluation of light intensity applied and the geometry of the light beam, likely towards a narrower geometry. At the same time, the relatively high ISO settings of typically 6400 to 10,000 required to achieve correct exposure are not optimal in terms of their sensor noise. Thus cameras of even higher sensitivity would be desirable, in order to employ less light. The minimum desired frame rate could be met, although the USB2 connection of the current camera model limits raw capture to 0.5 Hz.

The sample data indicate that the system is able to seamlessly survey flat seafloor for mosaicking and that the quality of the INS navigation data is good and in agreement with report of other authors [22]. The results of the photogrammetric reconstruction suggest that even subpixel registration of the images is feasible. Overall, this implies that the accuracy potential of the system is largely bound by the pixel footprint, which is 4 mm at 10 m altitude and 1.6 mm at 4 m altitude.

## 6. Conclusions

We have presented the technical layout and first results of a camera system for mosaicking and 3D reconstruction that is both capable of operating at advanced depths down to 6000 m and delivers color high-resolution imagery. Together with its carrier platform, the AUV ABYSS, it can deliver seamless seafloor coverage over large areas and currently ranks among the most capable systems available. Nevertheless, current challenges are presented by excess backscatter from the water column, which required the camera to be flown at its nominal lowermost range of 5 m above ground for consistent results. While the LED cluster has proven robust in terms of pressure tolerance, it is too exposed at the keel of the vehicle and negatively affects the hydrodynamic properties of the AUV. A redesign of the flash cluster geometry towards a narrower light cone and better protection is therefore under consideration.

Despite recent success during two expeditions surveying flat seafloors, we have not yet succeeded in demonstrating a case applied in rugged volcanic terrain. In order to do so, the system will be adapted to be flown on ROVs and hovering AUVs to minimize the risk of high velocity collisions. The current long vehicle turnaround time between dives must be shortened by implementation of intelligent data management and automatic image quality control during the mission, for which the system already has sufficient computing power. Another option could be the inclusion of structured light projectors (point and sheet lasers) to directly measure size and to derive high-resolution terrain data even under poor visibility conditions. A long-term goal is the abandonment of SLR technology back towards fully electronic imagers in order to exclude the possibility of mechanical failures.
